# TRANSITIONING OUT OF GRANDCHILDREN CAREGIVING: EFFECTS ON GRANDPARENTS’ EMOTIONAL WELL-BEING

**DOI:** 10.1093/geroni/igz038.1037

**Published:** 2019-11-08

**Authors:** Rita X Hu, Lydia Li, Toni C Antonucci

**Affiliations:** 1 University of Michigan, Ann Arbor, Ann Arbor, Michigan, United States; 2 University of Michigan, Ann Arbor, Michigan, United States

## Abstract

Research has suggested that grandparents caring for grandchildren experience both psychological gains and loss. Less clear is what happens to these grandparents after they exit from the caregiving role. This study used the Health and Retirement Study (HRS) 2010 to 2014 data to examine the effects of transitioning out of caregiving on the psychological well-being of grandparents. Psychological well-being was measured by the Positive and Negative Affect Schedule. We defined caregiving grandparents as grandparents who provide 100+ hours of care per year to their grandchildren. In the first wave, 8,278 respondents in the HRS were identified as caregiving grandparents. Among them, 3,914 continued to be caregivers and 4,364 transitioned out of the caregiving role by indicating they are no longer providing care in the second wave. Grandparents who transitioned out of caregiving are more likely to be older in age, less educated and not married. Linear regression analysis was conducted to compare the two groups (continuing vs. exiting caregiving) on positive and negative affect, controlling for the first wave’s measures of the dependent variable, sociodemographic characteristics and health status of respondents. Results show that grandparents who continued caregiving had less decline in positive affect than grandparents who transitioned out of caregiving (b = -0.05, SE = 0.02, p<0.01), adjusting for covariates. But they were not significantly different in negative affect. These findings indicate that older adults may have fewer sources of joy after exiting the role of caregivers of their grandchildren.

